# Emergence of G12 and G9 rotavirus genotypes in the Central African Republic, January 2014 to February 2016

**DOI:** 10.1186/s13104-017-3122-7

**Published:** 2018-01-05

**Authors:** Ulrich Aymard Ekomi Moure, Virginie Banga-Mingo, Jean Chrysostom Gody, Jason M. Mwenda, Jean Fandema, Diane Waku-Kouomou, Casimir Manengu, Thomas D’Aquin Koyazegbe, Mathew D. Esona, Michael D. Bowen, Ionela Gouandijka-Vasilache

**Affiliations:** 1 Ecole Doctorale Régionale D’AFRIQUE Centrale, Franceville, Gabon; 2 Institut Pasteur, Bangui, Central African Republic; 3 Complexe Pediatrique, Bangui, Central African Republic; 4 World Health Organization Regional Office for Africa, Brazzaville, Republic of Congo; 5 Centers for Disease Control and Prevention, Atlanta, GA 30329-4027 USA; 6 World Health Organization Country Office, Bangui, Central African Republic; 7 Present Address: World Health Organization Regional Office for Africa, Brazzaville, Republic of Congo; 8 Ministère de la Santé, de l’Hygiène et de la Population, Bangui, Central African Republic; 9 Present Address: World Health Organization Country Office, Bangui, Central African Republic

**Keywords:** Rotavirus, Genotype, Emergence, CAR

## Abstract

**Objectives:**

Rotavirus gastroenteritis is a major cause of death among children under 5 years globally. A rotavirus gastroenteritis surveillance program started in October 2011 in the Central African Republic (CAR) with the Surveillance Epidémiologique en Afrique Centrale (SURVAC) project. We present here genotyping results showing the emergence of G9 and G12 genotypes in Central African Republic.

**Results:**

Among 222 children hospitalized with acute gastroenteritis who had a stool sample collected at the sentinel site, Complexe Pédiatrique de Bangui (CPB), Bangui, Central African Republic, 100 (45%) were positive for rotavirus between January 2014 and February 2016. During this period the most common rotavirus strains were G1P[8] (37%), G12P[6] (27%) and G9P[8] (18%).

## Introduction

Rotavirus diarrhea is widespread with approximately 215,000 children less than 5 years of age dying each year due to severe dehydration caused by rotavirus [1]. Rotaviruses belong (RV) to the family Reoviridae, and the rotavirus genome consists of 11 double-stranded RNA gene segments that encode six structural (VP) and six non-structural proteins (NSP). Based on the two genes that encode the outer capsid proteins, VP4 (P-type) and VP7 (G-type), a widely used binary classification system was established for RV-A [2]. To date, at least 35 G and 50 P genotypes have been recognized in both mammalian and avian species. So far, 6 genotypes (G1P[8], G2P[4], G3P[8], G4P[8], G9P[8], and G12P[8]) are currently the most important genotypes in humans worldwide and are associated with 80–90% of the RVA associated disease burden [3–5]. In Africa and sub-Saharan Africa in particular there is a greater genetic diversity and emergence of new and unusual strains [6]. In addition to the 6 common strains mentioned above, rotavirus strains with unusual combination, such as G8P[6], G3P[4], G8P[4], G2P[6] as well as high levels of mixed infections and strains that cannot be assigned any specific genotype have been reported in sub-Saharan Africa [7].

In 2009, WHO recommended the introduction of rotavirus vaccine in all countries [8]. In the Central African Republic, the sentinel surveillance of rotavirus gastroenteritis was established in 2011 by the Ministry of Health, with the support of the Surveillance Epidémiologique en Afrique Centrale (SURVAC) Project [9]. The main objective of the project was to assess the burden of rotavirus gastroenteritis and identify rotavirus strains circulating in CAR before the introduction of a rotavirus vaccine which was initially scheduled for 2017. The SURVAC project completed in 2014 but the surveillance continues with support from the WHO, the Institut Pasteur de Bangui (IPB) and The Centers for Diseases Control and prevention (CDC). The surveillance results between 2011 and 2013 have been published [10]. We present here the rotavirus surveillance results for the period January 2014 to February 2016.

## Main text

### Methods

Stool samples were collected from children less than 5 years of age who met the WHO rotavirus gastroenteritis case definition at the sentinel site, the Complex Pédiatrique de Bangui (CPB), in the capital of CAR [11]. CPB is the only pediatric hospital of the country where, all severe diarrhea cases originated from Bangui and surroundings are referred.

At the sentinel site laboratory, the samples were first screened for group A rotavirus antigen by enzyme immunoassay (EIA) using the ProSpecT™ Rotavirus Microplate Assay (Oxoid, Ltd., Basingstoke, Hampshire, UK). Aliquots of all the samples were then stored at − 20 °C before transport to the Institut Pasteur de Bangui where results were confirmed by EIA using the same kit and genotyping assays performed as previously described [12]. Briefly, RNA extracts were subjected to multiplex semi-nested reverse transcription polymerase chain reaction (RT-PCR). Two genes; VP7 (896 bp) and VP4 (876 bp) were reverse-transcribed and amplified with primer pairs 9Con1-L/VP7-R and Con3/Con2, respectively [13, 14]. Reverse transcription of double strand RNA (dsRNA) was carried out with the OneStep RT-PCR Kit (Qiagen, Inc., Valencia, CA USA). After a 5 min denaturation at 97 °C, the RNA was mixed with kit reagents and incubated at 42 °C for 30 min to obtain complementary DNA (cDNA), immediately followed by the PCR reaction (30 cycles: 94 °C for 30 s; 42 °C for 30 s, 72 °C for 45 s; one cycle at 72 °C for 7 min). These first round RT-PCR products then were used in a semi-nested PCR (30 cycles, 94 °C for 45 s, 42 °C for 30 s 72 °C for 1 min; and 1 cycle at 72 °C for 7 min) to identify G types (G1, G2, G3, G4, G9 and G12) and P types (P[4], P[6], P[8]) [13, 14]. All PCR products were analyzed by electrophoresis in 2% agarose gels containing Gel Red (Biotium) and visualized under UV illumination. Sample of RNA extracts were sent to CDC for genotyping quality control and sequencing confirmation.

### Results and discussion

**Fig. 1:**
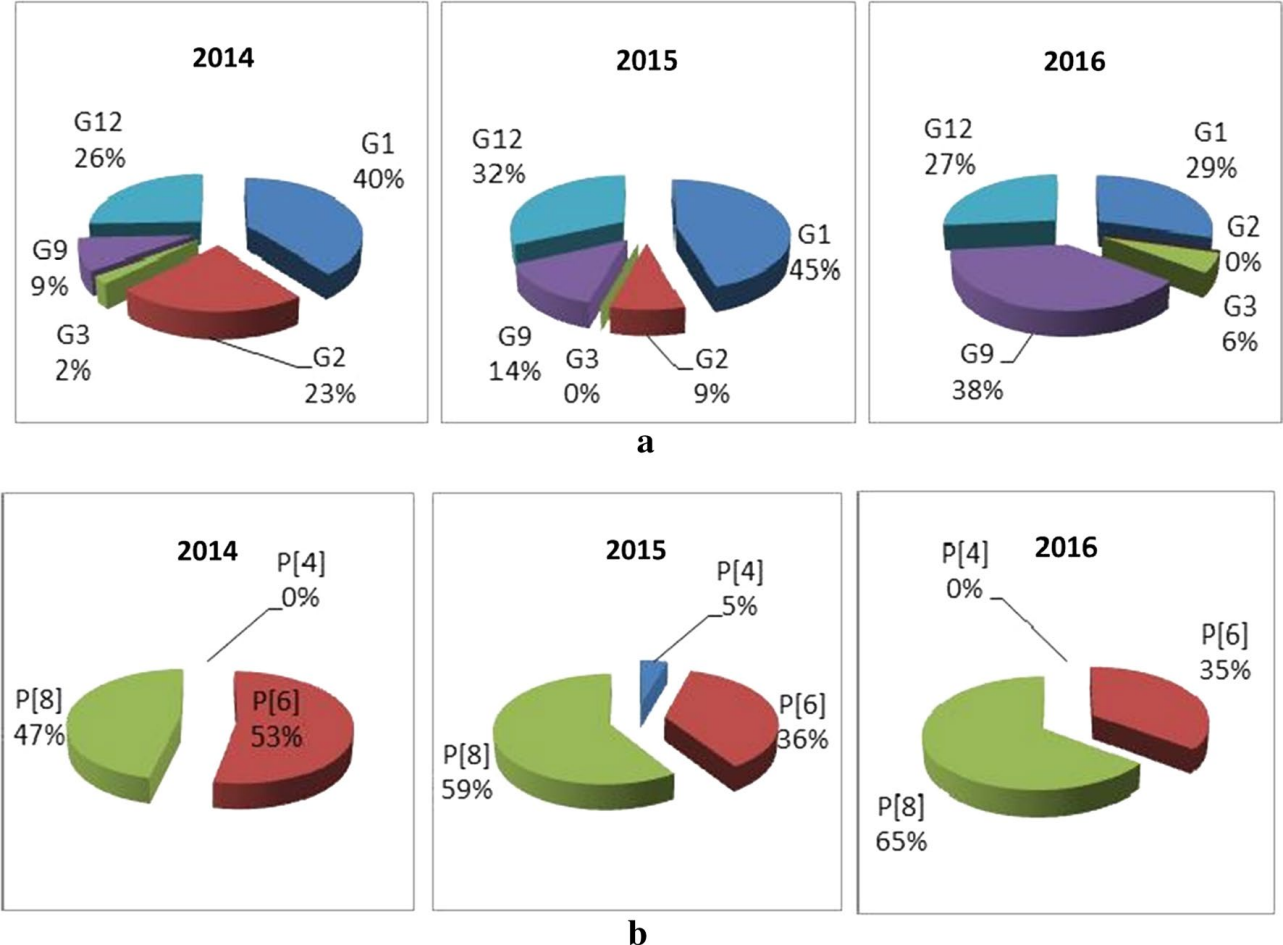
Annual G and P genotype distribution, January 2014–February 2016. **a** VP7(G) genotypes; **b** VP4 (P) genotypes. The total number of samples genotyped by year is 41, 22 and 37 in 2014, 2015 and 2016 (January to February only), respectively

**Fig. 2:**
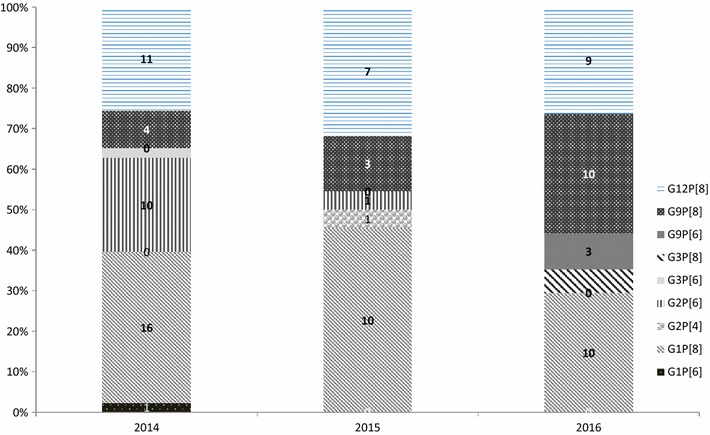
Frequency of Rotavirus G and P combinations in the Central African Republic, January 2014–February 2016. The total number of samples genotyped by year is 41, 22 and 37 in 2014, 2015 and 2016 (January to February only), respectively

Between January 2014 and February 2016, 222 stools samples were collected from children, with an 8 months mean age (range 1–55 months) and analyzed for detection of rotavirus antigen. The annual sample distribution was: 115 in 2014, 62 in 2015 and 45 from January to February 2016. Rotavirus was detected in 45% (100/222) of stool specimens by EIA and this prevalence is in agreement with the estimated 40% prevalence of rotavirus infection in other African countries [15] (Table 1). VP7 genotyping showed that G1 was the predominant strain 37% (37/100) followed by G12 and G9 with 27% (27/100) and 20%, (20/100), respectively, both of which have recently emerged in Africa [16, 17]. The other genotypes identified were G2 and G3 with 11% (11/100) and 3% (3/100), respectively. In a previous published study [10], G2 was found to be predominant in 66% (105/160) samples followed by G1, 28% (45/160) while G3 was not identified at all. In the same study the presence of G9 and G12 was for the first time reported at 3% (5/160) each. VP4 genotyping revealed that P[8] was predominant with 55% (55/100), followed by P[6] with 43% (43/100), compared to the 2011–2013 period when P[6] was predominant with 52% (83/106) with P[8] following at 35% (56/106). Two (2%) P[4] were identified, one of which was not confirmed by CDC. The annual distribution of G and P genotypes is shown in Fig. 1. The VP7/VP4 genotype combinations are shown in Fig. 2. G1P[8] was the predominant strain in 2014, 2015 and 2016 with 37.2% (16/43), 45.4% (10/22) and 29.4% (10/34), respectively. These data are consistent with our 2008 and 2011-2013 data and confirm data from other African countries [10, 18–20]. It was followed by G12P[6] with 25.5% (11/43), 31.8% (7/22) and 26.4% (9/34) in 2014, 2015 and 2016, respectively. G9P[8] was detected at 3% in 2013 [10], continued to circulate at 9.3% (4/43) in 2014, 13.6% (3/22) in 2015, and increased to 29.4% (10/34) in 2016. Other VP7/VP4 genotype combinations identified were: (i) G2P[6] which circulated in 2014 and 2015 at 23.2% (10/43) and 4.5% (1/22) respectively and 47% (75/106) during the 2011–2013 period; (ii) G1P[6] and G3P[6], which circulated in 2014 at 2.3% (1/43) each; (iii) G2P[4] which circulated in 2015 at 4.5% (1/22) and at 13% (21/106) from October 2011 to September 2013; and (iv) G9P[6] and G3P[8] with 8.8% (3/34) and 5.8% (2/34), respectively. Quality control was performed at the CDC on 102 samples, including two EIA negatives. The correlation of genotyping results between the two laboratories was 57%. Based on the low correlation between the genotyping results from CAR compared to results from CDC, the genotyping results reported‎ in this study were CDC results only. Due to the low degree of correlation, as a corrective action, a new set of primers from the CDC was sent to the CAR laboratory to help improve genotyping results. One of the samples bearing genotype P[4] was confirmed at the CDC, however, due to a sample leak during transportation, the tube containing the other sample arrived empty at the CDC and therefore could not be confirmed.

**Table 1:** Annual breakdown of stool samples tested for the presence of Rotavirus antigens by EIA (ProSpecT™) at Insttitut Pasteur de Bangui, Central African Republic (CAR), January 2014 to February 2016

Years	Rota positive (%)	Rota negative (%)	Total
2014^a^	41 (35.6)	74 (64.4)	115
2015^a^	22 (35.4)	40 (64.6)	62
2016^b^	37 (82.2)	8 (17.8)	45
Total	100 (45.0)	111 (55.0)	222

^a^CAR experienced military and political crises, especially in Bangui, where the civilian population remained cloistered at home for days to weeks

^b^January to February, high season for rotavirus transmission

### Conclusion

Genotyping data showed that genotypes G1P[8], G12P[6] and G9P[8] were the most common rotavirus strains circulating in Bangui during the study period. The detection of G12 and G9 strains contributes to the body of evidence that these genotypes are becoming increasingly dominant on the African continent and worldwide [15, 16, 21]. Currently, rotavirus vaccination has not been introduced into CAR; the introduction of Rotarix vaccine has been rescheduled for 2018. Rotarix vaccine has been shown to provide a good level of protection against severe rotavirus gastroenteritis in multiple African countries [22]. This vaccine is derived from a genotype G1P[8] strain but induces immunity against both homotypic and heterotypic rotavirus strains [22]. We therefore are expecting a drop in the number of gastroenteritis cases due to rotavirus infection after the introduction of the Rotarix vaccine [23]. Continued rotavirus surveillance is necessary to monitor the impact of the vaccine on the overall number of hospitalizations for severe gastroenteritis and for the emergence of new genotypes against which the available rotavirus vaccines may be less effective [24].

## Limitations

The study limitations are linked to the fact that CAR has only one sentinel site and did not reach the WHO recommendation to collect and test 250 samples per year. This is mainly due to the military and political problems that CAR has been experiencing since 2013.
